# Effect of massed v. standard prolonged exposure therapy on PTSD in military personnel and veterans: a non-inferiority randomised controlled trial

**DOI:** 10.1017/S0033291722000927

**Published:** 2023-07

**Authors:** Lisa Dell, Alyssa M. Sbisa, Andrew Forbes, Meaghan O'Donnell, Richard Bryant, Stephanie Hodson, David Morton, Malcolm Battersby, Peter W. Tuerk, Duncan Wallace, David Forbes

**Affiliations:** 1 Phoenix Australia – Center for Posttraumatic Mental Health, Department of Psychiatry, The University of Melbourne, Melbourne, Victoria, Australia; 2 School of Public Health and Preventive Medicine, Monash University, Melbourne, Victoria, Australia; 3 School of Psychology, University of New South Wales, Sydney, New South Wales, Australia; 4 Department of Veteran's Affairs, Canberra, Australian Capital Territory, Australia; 5 Department of Defence, Canberra, Australian Capital Territory, Australia; 6 College of Medicine and Public Health, Flinders University, South Australia, Australia; 7 Sheila C. Johnson Center for Clinical Services, Department of Human Services, University of Virginia, Charlottesville, Virginia, USA; 8 Australian Defence Force Center for Mental Health, Sydney, New South Wales, Australia

**Keywords:** Prolonged exposure therapy, military, PTSD, RCT, trauma, veteran

## Abstract

**Background:**

A short, effective therapy for posttraumatic stress disorder (PTSD) could decrease barriers to implementation and uptake, reduce dropout, and ameliorate distressing symptoms in military personnel and veterans. This non-inferiority RCT evaluated the efficacy of 2-week massed prolonged exposure (MPE) therapy compared to standard 10-week prolonged exposure (SPE), the current gold standard treatment, in reducing PTSD severity in both active serving and veterans in a real-world health service system.

**Methods:**

This single-blinded multi-site non-inferiority RCT took place in 12 health clinics across Australia. The primary outcome was PTSD symptom severity measured by the Clinician-Administered PTSD Scale for DSM-5 (CAPS-5) at 12 weeks. 138 military personnel and veterans with PTSD were randomised. 71 participants were allocated to SPE, with 63 allocated to MPE.

**Results:**

The intention-to-treat sample included 138 participants, data were analysed for 134 participants (88.1% male, *M* = 46 years). The difference between the mean MPE and SPE group PTSD scores from baseline to 12 weeks-post therapy was 0.94 [95% confidence interval (CI) −4.19 to +6.07]. The upper endpoint of the 95% CI was below +7, indicating MPE was non-inferior to SPE. Significant rates of loss of PTSD diagnosis were found for both groups (MPE 53.8%, SPE 54.1%). Dropout rates were 4.8% (MPE) and 16.9% (SPE).

**Conclusions:**

MPE was non-inferior to SPE in significantly reducing symptoms of PTSD. Significant reductions in symptom severity, low dropout rates, and loss of diagnosis indicate MPE is a feasible, accessible, and effective treatment. Findings demonstrate novel methods to deliver gold-standard treatments for PTSD should be routinely considered.

Posttraumatic stress disorder (PTSD) is a serious and disabling mental disorder, affecting military personnel and veterans at higher rates than the general community (McEvoy, Grove, & Slade, 2011; McFarlane & Hodson, 2011). Given PTSD is associated with long-term disability, impaired functioning, and high service use and healthcare costs (Sareen et al., 2007; Schnurr, Lunney, Bovin, & Marx, 2009; von der Warth, Dams, Grochtdreis, & König, 2020), it is imperative to offer effective treatments for PTSD that fit in with the work and life demands of those who need them.

Prolonged exposure (PE) therapy is a manualised first-line treatment for PTSD (Foa, Hembree, & Rothbaum, 2007; National Institute for Health and Care Excellence, 2018; Phoenix Australia, 2020), underpinned by emotion and information processing theories, and relies on fear extinction by engaging with the traumatic memory maintaining PTSD symptoms (Powers et al., 2015). A recent meta-analysis demonstrated the effectiveness of PE with treatment as usual/waitlist controls, showing a substantial benefit of PE with a standard mean difference of 1.51 (CI 95% 1.99–1.03) (Phoenix Australia, 2020). Despite the effectiveness of PE (Lewis, Roberts, Andrew, Starling, & Bisson, 2020), the 2–3 month duration of weekly standard PE (SPE) can be a practical barrier for implementation. Critically, in military members for whom adequate windows of availability in the same place may not exist, and for veterans with the potential for intervening major life events, several weeks to commit to and suitably engage in therapy is often not possible (Hall-Clark et al., 2019). Evidence of the effectiveness of massed forms of exposure therapies for PTSD (Wachen, Dondanville, Evans, Morris, & Cole, 2019) has driven interest in the feasibility of massed PE (MPE) therapy, whereby sessions are delivered once daily for 10 days. However, empirical evaluation of MPE is in its infancy and there is an urgent need to firmly establish its efficacy in a real-world clinical setting.

There is currently only one published RCT that provides early evidence for the equivalence of MPE to SPE (Foa et al., 2018). While scientifically strong, limitations of the study include that it was conducted from one US military clinical facility with only three therapists (Foa et al., 2018). Whilst the sample size was not insignificant, it must be highlighted that the generalisability of these findings to the ‘real-world’ are still unknown. Therefore, it is absolutely critical that a head-to-head randomised study comparing MPE to SPE is undertaken, which mimics the reality and diversity of the service system delivering clinical interventions. Secondarily, MPE must be examined within another geographical location and military population (including former serving members) to demonstrate generalisability.

The current RCT adheres to the rigour demanded by design (Dell et al., 2021), but makes important advances to the field of PTSD treatment due to being the first trial to implement MPE in a real-world health service environment by adopting a multi-site and multi-therapist approach. It was hypothesised that MPE would be non-inferior to SPE in reducing the severity of PTSD at 12 weeks post-treatment commencement.

## Methods

### Study design

This single-blinded multi-site non-inferiority RCT took place in 12 health clinics across eight sites within eight states and territories in Australia, and via telehealth during the COVID-19 pandemic. The Australian Defence Human Research Ethics Committee and Department of Veterans' Affairs Human Research Ethics Committee approved the protocol (now known as the Departments of Defence and Veterans' Affairs Human Research Ethics Committee), see Dell et al. (2021). The authors assert that all procedures contributing to this work comply with the ethical standards of the relevant national and institutional committees on human experimentation and with the Helsinki Declaration of 1975, as revised in 2008.

### Participants

Inclusion criteria were aged 18–80 years, current or former Australian Defence Force member, PTSD diagnosis as determined by the Clinician-Administered PTSD Scale for DSM-5 (CAPS-5), and a Criterion A trauma that occurred whilst serving. The index trauma did not need to be specific to deployment, and included events occurring on and off base and during training. The trial had a relatively high tolerance for including participants with mental health disorders comorbid to their PTSD, including moderate to severe depression and anxiety as well as moderate substance use problems. Exclusion criteria included current psychosis, mania, or high risk of harm to self or others, current severe alcohol or substance use disorder, and currently receiving other trauma-focused psychological therapy and unwilling/unable to pause. Participants were assessed for risk of harm to self or others at intake and baseline T1 assessment, and if clinically indicated during therapy and follow up assessment. Individuals were considered high risk of harm to self or others if they described active current plans and intent to harm or suicide. Severe alcohol and substance use disorders were assessed during intake and T1 assessment using the AUDIT and MINI, respectively. Participants who registered severe alcohol or substance use were not immediately excluded but informed that they could be re-assessed for suitability if they reduced their intake such that they could refrain from alcohol and substance use prior to assessment, and from the night before each therapy session. Participants taking psychotropic medication were required to be on a stable dose for the last four weeks and not intending to change for the duration of treatment. All participants provided informed written consent to participate in the trial.

## Procedures

### Assessment and randomisation

Participants underwent a pre-treatment baseline assessment (T1), during which an assessor administered a clinical interview and a self-report booklet. Eligible participants were randomly allocated in 1:1 ratio to SPE or MPE. Randomisation occurred using two components – randomisation to SPE or MPE, and then randomisation to a therapist to deliver the intervention. The random allocation of eligible participants to SPE or MPE was performed by a blinded research assistant (who was not involved in other aspects of the trial) using a computerised list created (by A.F.) using permuted blocks within each site. Second, random allocation to a therapist was made (by the blinded research assistant) using a separate randomisation list of therapists within each treatment condition within each site, designed to distribute the workload of therapists evenly over time within each site. Assessors (blinded to condition) conducted follow up assessments at 4 weeks post-treatment commencement (T2), and 12 weeks post-treatment commencement (T3). The success of blinding the assessors to condition was assessed by asking them to guess the condition that the participant was allocated to (in the 4-week follow up assessment). Based on their guesses, there was no evidence to suggest that the blinding had not been successful.

### Outcome measures

The primary outcome of this trial was posttraumatic stress symptom severity at 12 weeks (T3) as measured by the gold standard clinician-rated measure for PTSD, the CAPS-5. The structured clinical interview is comprised of 30-items scored on a 5-point Likert scale (0 = ‘absent’, 4 = ‘extreme/incapacitating’), measuring symptoms clusters of avoidance, negative alterations in cognition and mood, arousal and reactivity, and re-experiencing during the past month. The CAPS-5 provides an overall severity score ranging from 0–80, with moderate scores ranging from 23–34, severe scores between 35–47, and extreme ≥48. The CAPS-5 is one of the most widely used tools for diagnosing and measuring PTSD severity, with excellent reliability and validity (Weathers et al., 2018).

A number of other outcomes were assessed, including: self-report PTSD symptomatology (Posttraumatic Checklist for DSM-5) (Weathers et al., 2013); anxiety and depression (Hospital Anxiety and Depression Scale) (Zigmond & Snaith, 1983); problematic anger (Dimensions of Anger Reactions-5) (Forbes et al., 2014); quality of life (Assessment of Quality of Life Scale-6 dimension version) (Richardson et al., 2012); disability (World Health Organization Disability Assessment Schedule 2.0) (WHODAS Group, 2000); and alcohol use (Alcohol Use Disorders Identification Test) (Babor, de la Fuente, Saunders, & Grant, 1989). Participant 12-month follow-up data collection is ongoing and long-term PTSD outcomes, in addition to the results of secondary measure outcomes, will be reported in future publications.

A Data and Safety Monitoring Board was established for consideration of stopping or modifying the trial for safety reasons. Interim analysis was conducted when approximately 50% of the sample was recruited. The Board recommended the continuation of the trial. There were no clinically significant adverse events reported throughout the trial.

### Treatment intervention

The SPE group received 10 weekly sessions of 90-min face-to-face manualised therapy (Foa et al., 2007) and the MPE group received identical therapy delivered rapidly over 2 weeks (Dell et al., 2021). Both groups undertook *in vivo* homework activities. Telehealth treatment (offered during COVID-19 restrictions) involved the same format and duration of therapy but took place using online video platforms. Participants were considered treatment completers if they attended at least seven sessions, consistent with previous PE research (Sripada & Rauch, 2015; Tuerk et al., 2013; Yoder, Tuerk, & Acierno, 2010), considered a withdrawal if they were randomised but did not commence treatment, and considered a dropout if they commenced treatment but discontinued. Both participants that withdrew or dropped out of the trial were classified as treatment non-completers.

For both groups, therapists made phone contact with the participant one-, three- and six-weeks post-therapy to encourage the participant to continue undertaking *in vivo* activities and to monitor activity, as is a common practice in disseminated settings.

### Therapist training and supervision

Thirty-eight therapists underwent 4-day credentialed PE training facilitated by an international expert (P.T.), who has served as a national trainer for the United States Department of Veterans Affairs' and the Department of Defense. Nineteen of the aforementioned therapists also completed training for PE via telehealth with the same trainer. Therapists attended fortnightly supervision to prevent drift from protocol, and therapy sessions were audio recorded for the purpose of monitoring treatment fidelity (Dell et al., 2021). Fidelity checks were conducted by an independent expert on every session of a therapist's first participant, on session one and three for a therapist's second participant, and then on a random sample of 10% of cases.

### Sample size

Power and sample size were based on the upper endpoint of the 95% confidence interval (CI) for the difference in mean change scores from baseline to 12 weeks between MPE and SPE (adjusted for baseline) being less than the margin of 7 CAPS-5 points. The margin of 7 points was drawn from the extant randomised-controlled study data examining evidence-based PTSD treatment, which varies in its minimal change range from 7 to 10 CAPS-5 points (Cloitre et al., 2010; Varker et al., 2020). The margin of 7 therefore represented the most conservative estimate of minimal change reported in the literature, with a between-subject s.d. of 15.6 points and a baseline-to-12-week correlation in CAPS-5 scores of 0.32, a total of 120 completed participants has 73% power to detect non-inferiority when MPE is truly non-inferior, and with a false positive error rate (i.e. falsely declaring non-inferiority) of at most 2.5% (Dell et al., 2021).

### Statistical analysis

Baseline (T1) characteristics of the randomised groups were compared using descriptive statistics. CAPS-5 scores over time were reported graphically and with descriptive statistics. Analyses of CAPS-5 scores at 12 weeks (T3) was performed using linear regression with change from baseline as the dependent variable and adjusting for the baseline value of CAPS-5 score (i.e. ANCOVA). Multiple imputations of missing CAPS-5 scores at T2 (MPE 15.9%; SPE 26.8%) and T3 (MPE 22.2%; SPE 28.2%) was performed using full information maximum likelihood estimation procedure with variables in the imputation model chosen if their correlation with CAPS-5 score or with an indicator of CAPS-5 score being missing was 0.40 or larger (Graham, 2012). Imputations were conducted separately for each treatment group to produce 10 imputed datasets, with results combined across imputations using Rubin's rules. Non-inferiority was indicated if the upper endpoint of the 95% CI computed using multiple imputations for the difference in mean CAPS-5 scores (MPE-SPE) lay below 7. No assessment of heterogeneity of treatment effect across sites was performed due to the expectation of homogeneity arising from the standardisation of treatment across sites, alongside supervision and fidelity monitoring.

Whilst not included in the original protocol (Dell et al., 2021), but given it is routinely reported, prior to any data analysis there was a decision to assess for non-inferiority in loss of diagnosis. The pooled estimates of the frequencies of CAPS-5 diagnoses, resulting from an imputation process similar to that utilised for CAPS-5 total scores, were used except that a logistic, rather than linear, a function was used to predict CAPS-5 diagnosis for missing data.

All analyses were performed in SPSS v27.

## Results

**Fig. 1.**
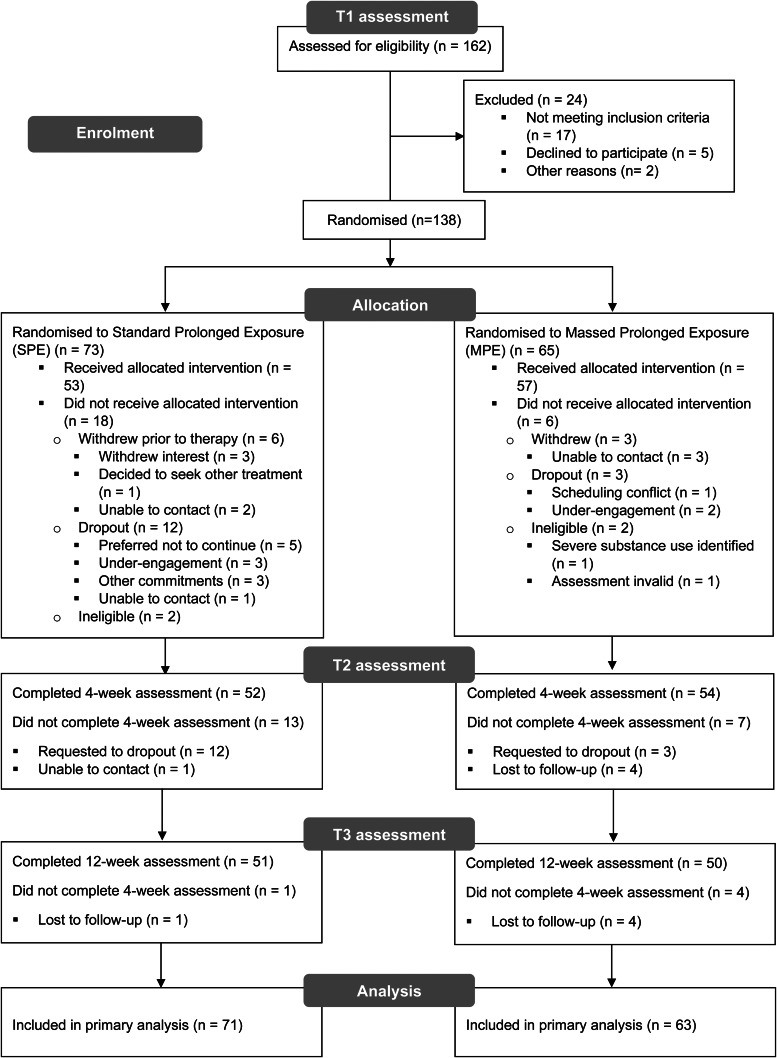
CONSORT diagram describing flow of participants through the study. CONSORT = Consolidated Standards of Reporting Trials; Withdrew = participants who were randomised to a group but did not commence treatment; Dropout = participants who were randomised to a group, commenced treatment but discontinued.

One hundred and sixty-two individuals were assessed for eligibility at T1 (Fig. 1). Twenty-four individuals were ineligible for randomisation due to the following reasons: not meeting inclusion criteria (e.g. no diagnosis of PTSD; severe substance use), declined to participate (e.g. decided to undertake other trauma-focussed therapy; did not return attempts to contact), and other reasons including relocation that was too far from trial sites. Of the 138 who were found to be eligible and initially randomised to therapy, four were later found to be ineligible and were excluded from the intention-to-treat sample. The final intention-to-treat sample comprised 134 participants who were randomised to MPE (*n* = 63) or SPE (*n* = 71). Recruitment occurred between September 2016 – October 2020 and ceased once study funding was exhausted. Follow up assessments occurred until January 2021. One hundred and ten participants completed therapy (≥7 of 10 sessions), 9 withdrew from the trial, and 15 participants dropped out.

The sample was predominately male (*n* = 118; 88.1%), with the majority aged between 28–57 years (*M* = 46 years), and 66.4% of participants reported they were an ex-serving military member. The majority of participants had symptoms of depression (81.1%; *n* = 107) and anxiety (88.0%; *n* = 117), and 51 (38.1%) had probable alcohol or substance use disorder (see Table 1 for sample demographics and service characteristics). During the intake assessment, 62.7% (*n* = 84) of the sample reported a low or medium risk of self-harm. At T1, all participants had a diagnosis of PTSD on the CAPS-5. Therapist adherence to the protocol was rated by an independent expert and adherence was 94.0%.

**Table 1.** Sample demographic and service characteristics (*n* = 134)

	MPE (*n* = 63)		SPE (*n* = 71)	
	
	*N*	%	*N*	%
Sex				
Male	56	88.89	62	87.32
Female	7	11.11	9	12.68
Age, years (*M*, s.d.)	44.29	10.83	46.69	12.68
Age, categories				
18–27	3	4.76	3	4.23
28–37	13	20.63	18	25.35
38–47	23	36.51	16	22.54
48–57	17	26.98	22	30.99
58+	7	11.11	12	16.90
Education				
Primary/secondary school	17	27.42	24	34.29
Certificate/diploma	32	51.61	27	38.57
University	13	20.97	19	27.14
Serving status				
Current ADF member	21	33.33	24	33.80
Ex-serving	42	66.67	47	66.20
Service				
Navy	14	22.95	18	25.35
Army	37	60.66	43	60.56
Air force	9	14.75	9	12.68
Other non-Australian military	1	1.64	1	1.41
Rank				
Commissioned officer	11	18.03	11	15.71
Non-commissioned officers	36	59.02	38	54.29
Other ranks	14	22.95	21	30.00
Time served (years) (*M*, s.d.)	15.35	10.30	15.19	10.72
Time served, categories				
0–4	9	14.75	9	13.43
5–9	15	24.59	19	28.36
10–19	15	24.59	21	31.34
20+	22	36.07	18	26.87
Ever deployed				
No	5	8.06	10	14.08
Yes	57	91.94	61	85.92
Number of deployments (*M*, s.d.)	5.00	7.46	3.73	5.45
Number of deployment exposures (*M*, s.d.)				
Traumatic	8.40	3.44	7.39	3.30
Environmental	5.31	0.88	5.02	1.21
Other				
HADS depression 8+	50	81.97	57	80.28
HADS anxiety 8+	57	91.94	60	84.51
AUDIT 8+	37	60.66	42	63.64
MINI alcohol use disorder	18	28.57	27	38.03
MINI substance use disorder	5	7.93	1	1.40
Number of psychotropic medications in use				
None	19	30.16	34	47.89
1	15	23.81	18	25.35
2	14	22.22	6	8.45
≥3	15	23.81	13	18.31

*n*, sample size; *M*, mean; s.d., standard deviation.

*Note*. All data was collected via participant self-report during the T1 assessment with the exception of alcohol use disorder and substance use disorder, which were assessed using the clinician-administered Mini International Neuropsychiatric Interview (MINI) and corresponds to the previous 12 months. HADS scores 8+ reflect individuals who registered mild to severe symptoms of anxiety or depression; AUDIT scores 8+ reflect individuals who registered harmful/hazardous drinking or alcohol dependence.

Interim analysis by the Data and Safety Monitoring Board examined unblinded data for *n* = 52 participants. Linear regression analysis, with treatment condition as the factor, and baseline scores as the covariate were conducted. Two-sided repeated asymmetric CIs were presented to assess non-inferiority. Results indicated no significant differences in CAPS-5 scores at 12-weeks post-treatment commencement and the boundaries of the CIs did not exceed the threshold established to declare non-inferiority or harm; *F*(2, 33) = 4.18, *p* = 0.024, *R*^2^ = 0.20, *R*^2^adjusted = 0.15.

**Fig. 2.**
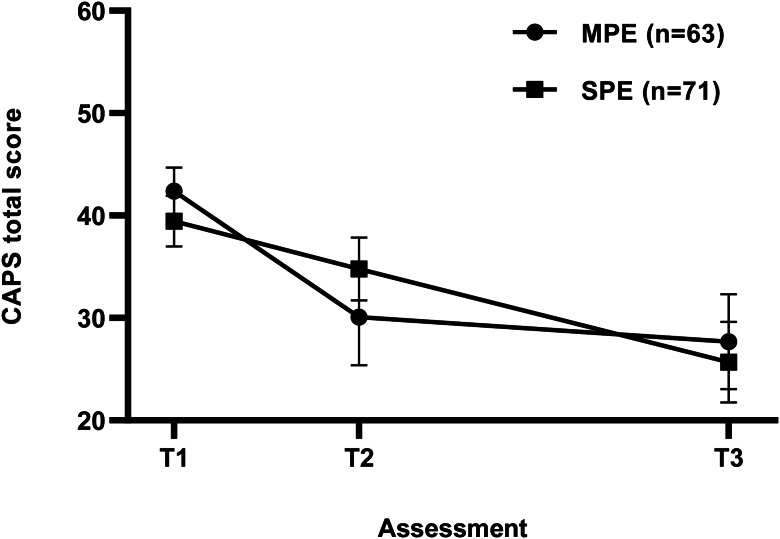
Mean clinician-administered posttraumatic stress disorder scale for DSM-5 (CAPS-5) scores at T1 (baseline), T2 (4 weeks post-commencement of therapy), and T3 (12 weeks post-commencement of therapy). MPE = Massed prolonged exposure (*n* = 63), SPE = Standard prolonged exposure (*n* = 71). Error bars indicate 95% CIs.

**Fig. 3.**
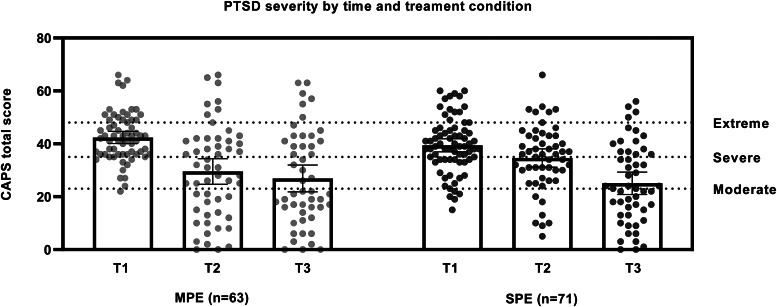
PTSD severity as measured by the clinician-administered posttraumatic stress disorder scale for DSM-5 (CAPS-5) at T1 (baseline), T2 (4 weeks post-commencement of therapy), and T3 (12 weeks post-commencement of therapy) including individual data points and CAPS-5 severity categories: moderate (scores 23–34); severe (35–47); extreme (48–80). MPE = Massed prolonged exposure (*n* = 63); SPE = Standard prolonged exposure (*n* = 71). Error bars indicate 95% CIs.

As outlined in the data analysis section, all analyses reported subsequently here were conducted on the intent-to-treat sample. For the MPE and SPE groups, CAPS-5 scores reduced (meaning symptoms decreased) by 14.69 and 13.75 points, respectively, from baseline [MPE (*M* = 42.38, s.d. = 9.18) and SPE (*M* = 39.48, s.d. = 10.43)] to 12 weeks post-treatment [MPE (*M* = 27.69, s.d. = 18.42) and SPE (*M* = 25.68, s.d. = 16.59)] (Fig. 2 represents multiply imputed means). The multiply-imputed estimate of the difference between the MPE and SPE group means was 0.94, with 95% CI −4.19 to +6.07. The upper endpoint of the 95% CI was below the value of +7, indicating that the MPE group was non-inferior to the SPE group. Phrased in terms of Cohen's d, this result is an effect size of 0.054 with 95% CI −0.24 to +0.34. Fig. 3 provides visualisation of the individual CAPS-5 data points and severity categories across time.

There were no adverse events throughout the trial.

There were more non-completers in the SPE group (*n* = 18/71, 25.4%) than in the MPE group (*n* = 6/63, 9.5%); this number includes individuals who were randomised and did not commence session 1, and individuals who dropped out during therapy. Three (4.8%) MPE participants and twelve (16.9%) SPE participants commenced therapy then dropped out prior to completion, which was significantly different between groups (χ^2^ (1) = 5.35, *p* = 0.021). There were no significant differences between completers and non-completers for the MPE or SPE groups on gender, education level, service type, rank, time served, deployment status and frequency, and PTSD. However, for the MPE group non-completers were significantly (*p* = 0.015) younger (*M* = 34.17; s.d. = 8.95) than completers (*M* = 45.35; s.d. = 10.51).

In post-hoc assessment, 46.7% of the MPE group had no PTSD diagnosis at 4 weeks, as did 40.7% of the SPE group, however this difference was not statistically significant; *p* = 0.441. At 12 weeks, the proportion of those without a diagnosis in the MPE group (53.8%) was equivalent to the SPE group (54.1%), *p* = 0.959.

## Discussion

This multi-site and multi-therapist RCT investigated whether MPE was non-inferior to SPE in reducing the severity of PTSD symptoms in military personnel and veterans. As hypothesised, MPE delivered across 2 weeks was non-inferior to SPE delivered weekly for 10 weeks in reducing clinician-rated PTSD symptom severity at 12 weeks post-treatment. This is the first large-scale trial of PE conducted in an Australian military and veteran population, and the first-ever real-world RCT to integrate MPE across clinical sites within a health service system. The specific design of this RCT increases the generalisability of the findings, and importantly, this trial challenges a perception that exposure therapy for trauma is too distressing for both the patient and therapist (Ruzek et al., 2016), with low rates of dropout once therapy has begun and positive gains made for most participants.

The result of non-inferiority indicates that MPE is a promising approach for PTSD treatment for military personnel and veterans, who are often challenged to commit large blocks of time for therapy. Particularly noteworthy are the low rates of dropout across the two conditions, with that of the MPE group (4.8%) 3.5 times less than the SPE group (16.9%), especially in light of the recent RCT comparing prolonged exposure therapy to cognitive processing therapy where dropout by the group were 55.8% and 46.6% respectively (Schnurr et al., 2022). It has been argued that MPE can address the distraction, avoidance, and de-motivation that commonly occurs between therapy sessions (Sherrill et al., 2020), and it is possible that the reduced time commitment required could circumvent a significant proportion of potential drop out from evidence-based therapy. It is also plausible that by engaging daily with a therapist there is a greater sense of support, and therefore commitment, to the process. Indeed, recent qualitative research has shown that US veterans believe the structure of MPE both limits distractions and avoidance, and reinforces engagement and enhances motivation (Sherrill et al., 2020).

If the outcomes of reduced dropout from this study are replicated, the finding would represent a very significant step forward in evidence-based treatment research, as reducing dropout across evidence-based trauma-focused treatment modalities has been a highly sought after yet elusive goal for the field (Varker et al., 2021).

Study conclusions should be considered in relation to limitations. Firstly, the population of interest in this study was military and veteran and it is therefore unclear whether similar outcomes would be found in other PTSD affected populations. Secondly, the sample was predominantly male, which although reflective of the gender differential in most military and veteran populations, means it is unclear whether these results can generalise to all-female military/veteran members. Finally, whilst the briefness of MPE may be advantageous for some, for others is may be a challenge to engage in therapy for 10 days across two weeks. Future analysis will examine change over time in the other outcome measures (such as self-reported PTSD, anxiety and depression) and the maintenance of symptom reduction through longer-term follow up with trial participants. Future research should consider replicating this study in other populations and in a more gender-balanced military study.

In veterans and military personnel with trauma occurring during service, MPE can clearly provide equivalent reductions in PTSD symptom severity as SPE. The implications of this finding for clinicians and researchers are significant and represents an exciting advancement in our understanding of treatment engagement and dropout reduction, and that is, that rapid evidenced-based therapy which relies on frequent contact and active engagement between therapist and client may indeed serve as a mechanism for keeping clients in therapy long enough to receive the full dose of treatment.

Equally important is that individuals may feel more empowered if given options for engaging in therapy that can be delivered flexibly around the demands of life and work for those in, and out of, the military. Empowering individuals leads to better engagement, and has been shown to be associated with improved PTSD outcomes and improved satisfaction with treatment decisions (Stacey et al., 2017; Watts et al., 2015).

As the first-ever multi-site and multi-therapist massed and standard PE trial, this RCT has high ecological validity, using the exact settings where Australian military members and veterans receive therapy, and as such, represents a significant contribution to our understanding of how to best treat PTSD. This trial has demonstrated that in a real-world setting, MPE is non-inferior to SPE in treating military members and veterans with PTSD, it has advanced our understanding of treatment dropout and importantly has served to confirm that there are options for individuals in the way they engage in trauma-focused, exposure-based psychological therapy.
